# Typing of biliary tumor thrombus influences the prognoses of patients with hepatocellular carcinoma

**DOI:** 10.20892/j.issn.2095-3941.2020.0202

**Published:** 2021-08-15

**Authors:** Juxian Sun, Jiayi Wu, Chang Liu, Jie Shi, Yonggang Wei, Jianyin Zhou, Zhibo Zhang, Wan Yee Lau, Maolin Yan, Shuqun Cheng

**Affiliations:** 1 Department of Hepatic Surgery VI, The Eastern Hepatobiliary Surgery Hospital, Second Military Medical University, Shanghai 200433, China; 2 Shengli Clinical Medical College, Fujian Medical University, Fuzhou 350001, China; 3 Department of Hepatobiliary Surgery, Fujian Provincial Hospital, Fuzhou 350001, China; 4 Department of Hepatobiliary Surgery, West China Hospital, Sichuan University, Chengdu 610041, China; 5 Department of Hepatobiliary Surgery, Zhongshan Hospital, Xiamen University, Xiamen 361004, China; 6 Department of Hepatobiliary Surgery, The First Affiliated Hospital of Fujian Medical University, Fuzhou 350002, China; 7 Faculty of Medicine, The Chinese University of Hong Kong, Hong Kong 999077, China

**Keywords:** Hepatocellular carcinoma, biliary tumor thrombus, staging system, surgery, prognosis

## Abstract

**Objective:**

To establish a new classification of biliary tumor thrombus (BTT).

**Methods:**

Overall survival of patients with BTT was first used to determine whether it correlated with current hepatocellular carcinoma staging systems. Univariate and multivariate analyses were used to determine factors affecting the overall survival (OS) to form the basis of our new classification for BTT.

**Results:**

All 6 international staging systems showed overlapping survival curves. Univariate followed by multivariate analyses showed that total bilirubin and intrahepatic/extrahepatic BTT were significant risk factors of OS. Based on these data, a new BTT classification was defined as: Type I: intrahepatic BTT; and Type II: extrahepatic BTT involving a common bile duct or common hepatic duct. Type I was further subdivided into type Ia: BTT involving a second-order intrahepatic duct or above, and type Ib: BTT involving a first-order intrahepatic duct. Type II was further subdivided into type IIa and type IIb using a cut-off total bilirubin (TB) > 300 μmol/L. The numbers (percentages) of patients with types I and II BTT were 69 (34.2%) and 133 (65.8%), respectively. The median OS of type I patients was significantly higher than that of type II patients (37.5 months *vs.* 23.2 months; *P* = 0.002). Using subgroup analyses, OS outcomes were significantly different between the subgroups of type IIb and type IIa, although there was no significant difference between the type Ia and type Ib subgroups (*P* = 0.07).

**Conclusions:**

A new BTT classification was established to predict prognoses of HCC patients with BTT who underwent liver resection.

## Introduction

Hepatocellular carcinoma (HCC) is the fifth most common neoplasm and the third leading cause of cancer-related mortality worldwide^1^. Although HCC has a strong tendency to involve vascular structures, biliary tumor thrombus (BTT) is also a well-known and important clinical presentation. The incidence of BTT has been reported to be 1%–9%^2–4^. Because jaundice presents in a significant percentage of HCC patients with BTT at the time of diagnosis, misdiagnosis as progressive liver failure or diffuse tumor infiltration of the liver parenchyma often occurs. With improvements in imaging techniques, the diagnosis of BTT is becoming more straightforward and accurate^5^. Clinically, it is important to identify this group of patients because surgical treatment can be beneficial^6^. A Korean-Japanese multicenter study^2^ showed a 5-year survival of up to 43.6%, indicating that an aggressive surgical approach is the choice of treatment in selected BTT patients.

The current international HCC staging systems that are commonly used, including the TNM classifcation^7^, Cancer of the Liver Italian Program (CLIP) score^8^, the Japan Integrated Staging (JIS) scoring system^9^, and the Barcelona Clinic Liver Cancer (BCLC) classification^10^, only consider vascular invasion to be an important prognostic factor, but do not include BTT. Several studies have classified BTT types^11,12^, but all these classifications are based only on anatomy, which is inadequate to predict the prognosis and guide management. A specific classification that includes clinical parameters in addition to anatomy is urgently needed. This study therefore established a new classification to better stratify and refine the types of BTT, with the aim of improving prognostic predictions and guiding clinical management.

## Materials and methods

### Patients

A multicenter retrospective study was conducted on a prospectively maintained clinical and pathological database of HCC patients. Consecutive HCC patients with BTT who underwent liver resection at the Eastern Hepatobiliary Surgery Hospital (EHBH), Fujian Provincial Hospital, West China Hospital of Sichuan University, First Affiliated Hospital of Fujian Medical University, and Zhongshan Hospital of Xiamen University were enrolled in this study. The presence of BTT was diagnosed using preoperative radiological imaging (ERCP/CT/MRI/ultrasound) and was subsequently confirmed by postoperative histopathological studies^3,5^. The inclusion criteria were as follows: (1) age between 18–75 years with an Eastern Cooperative Oncology Group performance status score of 0 or 1; (2) resectable HCC, which was defined as either a single tumor <10 cm in diameter or multiple HCCs confined to 1 hemi-liver; (3) macroscopic BTT on preoperative medical imaging; (4) no extrahepatic or distant metastases; and (5) no other associated malignancies. The present study was approved by the Research Ethics Committees of all participating hospitals. Informed consent was obtained from all patients prior to treatment and for their data to be used for research purposes.

All patients underwent routine preoperative imaging, including abdominal ultrasonography, contrast-enhanced magnetic resonance imaging (MRI) and/or computed tomography (CT) of the abdomen and chest. Routine serological examinations included complete blood analyses, liver and renal function, hepatitis B and C serology, HBV DNA load, and serum α-fetoprotein (AFP) level tests.

### Surgical procedures

All the preoperatively and intraoperatively detected lesions were resected. If the BTT was located within the planned hepatic resection region, the BTT was removed *en bloc* with the resected part of the liver. If the BTT extended to the common bile duct, thrombectomy with extraction of the BTT from the opened stump of the right/left hepatic duct was performed in combination with cholangio-jejunostomy. Intraoperative ultrasound was used to confirm that no residual tumor or BTT was left behind.

### Follow-up

All patients were followed-up once every month for the first 3 months postoperatively and then once every 3 months thereafter. The follow-up examinations included routine blood tests, liver function tests, AFP tests, chest radiography, abdominal ultrasound, and CT/MRI. HCC recurrence was diagnosed based on typical imaging features on CT and/or MRI, with or without abnormal AFP levels. If HCC recurrence was diagnosed, appropriate treatments including further liver re-resection, local ablative therapy, and/or regional or systematic therapy were performed.

### Statistical analysis

Categorical variables were analyzed using the χ^2^ test or Fisher’s exact test, and continuous variables are expressed as the mean ± standard deviation (SD) and were compared using the Mann-Whitney test. Survival curves of recurrence-free survival and OS were obtained using the Kaplan-Meier method and compared using the log-rank test. Median survival times and their 95% confidence intervals (CIs) were reported. Factors that were found to be significantly (*P* < 0.05) associated with survival using univariate analysis and any imbalanced factors between groups were entered into a Cox A proportional hazards model to test for significant effects while simultaneously adjusting for multiple factors. For all tests, a *P* < 0.05 was considered statistically significant. All statistical analyses were performed with SPSS statistical software for Windows, version 24.0 (Chicago, IL, USA).

## Results

### Patient characteristics

This study included 202 consecutive HCC patients with BTT who underwent liver resection from June 2009 to December 2017 at the Eastern Hepatobiliary Surgery Hospital, Fujian Provincial Hospital, West China Hospital of Sichuan University, First Affiliated Hospital of Fujian Medical University, and Zhongshan Hospital of Xiamen University. Among the 202 patients, 41 (20.3%) patients had vascular invasion, and 10 (5.0%) patients had lymph node metastases. The numbers (percentages) of patients with intrahepatic and extrahepatic BTT were 146 (34.2%) and 133 (65.8%), respectively. At a median follow-up of 28.4 months (range, 13–112 months), 141 patients (69.8%) died.

### Predictive accuracy of the commonly used international staging systems

**Figure 1:**
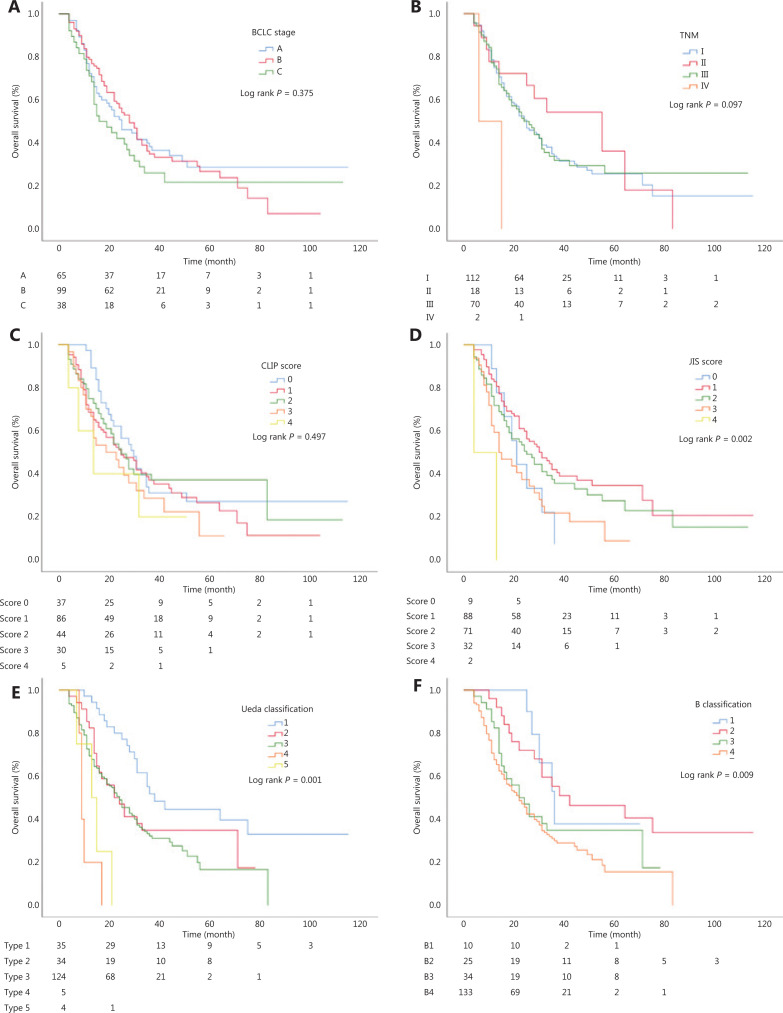
Kaplan-Meier curves of 6 systems which all showed overlapping survival curves. (A) Kaplan-Meier curves of the Barcelona Clinic Liver Cancer staging. (B) Kaplan-Meier curves of the TNM staging. (C) Kaplan-Meier curves of the CLIP. (D) Kaplan-Meier curves of the JIS. (E) Kaplan-Meier curves of the Ueda classification system. (F) Kaplan-Meier curves of the Japanese B-classification system. All 6 systems showed overlapping survival curves.

Kaplan-Meier curves were generated for the BCLC staging, TNM staging, CLIP, JIS, Ueda, and Japanese B-classification systems (**Figure 1**). The survival outcomes are also shown in **Table 1**. All 6 systems showed overlapping survival curves, with no clearly defined prognostic strata.

**Table 1:** Survival outcomes of the 6 staging systems

Current model	*n*	1-year survival	3-year survival	5-year survival	*P*
TNM					0.097
1	112	75.0%	28.6%	9.8%	
2	18	77.8%	44.4%	11.1%	
3	70	75.7%	24.3%	10.0%	
4	2	50.0%	0	0	
JIS score					0.002
0	9	88.9%	0	0	
1	88	83.0%	35.2%	12.5%	
2	71	71.8%	26.8%	9.9%	
3	32	59.4%	21.9%	3.1%	
4	2	50.0%	0	0	
CLIP score					0.497
0	37	97.3%	30.6%	13.5%	
1	86	68.6%	37.3%	10.5%	
2	44	75.0%	48.5%	9.1%	
3	30	70.0%	33.3%	3.3%	
4	5	60.0%	33.3%	0	
BCLC stage					0.375
A	65	72.3%	35.4%	10.8%	
B	99	78.8%	25.3%	9.1%	
C	38	71.1%	23.7%	7.9%	
B-classification					0.009
1	10	100.0%	20.0%	10.0%	
2	25	96.0%	52.0%	32.0%	
3	34	82.4%	32.4%	23.5%	
4	133	67.7%	23.3%	1.5%	
Ueda-classification					0.001
1	35	97.1%	42.9%	25.7%	
2	34	82.4%	32.4%	23.5%	
3	124	69.4%	25.0%	1.6%	
4	5	20.0%	0	0	
5	4	25.0%	0	0	

Multivariate analysis showed that TB < 300 μmol/L hazard ratio (HR: 2.075; 95% CI; 1.210–3.559; *P* = 0.008) and intrahepatic/extrahepatic BTT (HR: 1.600; 95% CI, 1.097–2.332, *P* = 0.015) were significant risk factors for OS (**Table 2**).

**Table 2:** Univariate analysis and multivariate analysis of overall survival

Variable	Univariate analysis			Multivariate analysis		
	*β*	95%CI	*P*	*β*	95%CI	*P*
Age (years) < 50	0.003	1.003 (0.714–1.409)	0.085			
Gender (*N*), male: female	0.131	1.140 (0.710–1.829)	0.589			
AFP (μg/L), < 400: ≥ 400	−0.195	0.823 (0.589–1.150)	0.254			
ALT (U/L), < 40: ≥ 40	−0.025	0.976 (0.674–1.413)	0.897			
Total bilirubin (μmol/L) > 300	0.905	2.472 (1.461–4.183)	0.001	0.73	2.075 (1.210–3.559)	0.008
Hbs Ag (*N*), positive: negative	0.079	1.082 (0.681–1.721)	0.738			
HBV-DNA (copies/mL) < 1,000	0.124	1.122 (0.764–1.680)	0.536			
Tumor number, single	0.055	1.056 (0.740–1.508)	0.764			
Tumor diameter, < 5 cm	−0.039	0.962 (0.686–1.348)	0.821			
Coexistence of PVTT	0.378	1.460 (0.983–2.167)	0.061			
Child-Pugh A:B	0.434	1.543 (1.106–2.154)	0.011	0.066	1.068 (0.700–1.631)	0.759
ALP < 135 U/L	0.215	1.240 (0.870–1.766)	0.234			
GGT < 45 U/L	1.28	3.596 (0.887–14.576)	0.073			
PT < 13 S	0.207	1.230 (0.857–1.766)	0.261			
BDTT, Intrahepatic: extrahepatic	0.555	1.743 (1.207–2.516)	0.003	0.47	1.600 (1.097–2.332)	0.015
Anatomic: local resection	0.32	1.377 (0.989–1.918)	0.058			

### Survival analysis of our new classification of BTT

**Figure 2:**
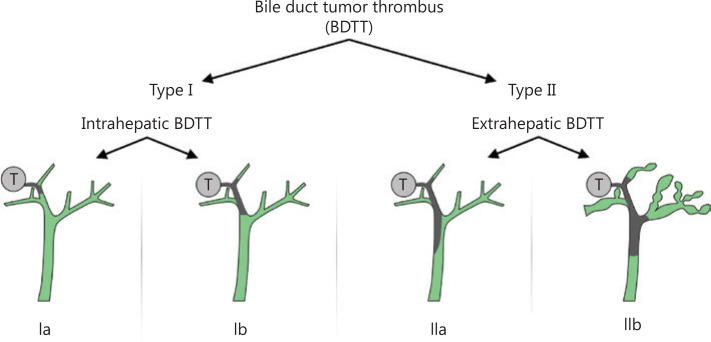
Patterns of biliary tumor thrombus type systems. T: hepatic tumor.

HCC patients with BTT were divided into 2 grades based on the 2 significant risk factors for OS of TB and intrahepatic/extrahepatic BTT: type I, intrahepatic BTT; type II, extrahepatic BTT involving the common bile duct or common hepatic duct. Type I was further subdivided: type Ia, BTT involving the second-order intrahepatic duct or above; and type Ib, BTT involving the first-order intrahepatic duct. Based on the need for preoperative biliary drainage for patients with TB > 3,000 μmol/L, type II was further subdivided into type IIa and type IIb (**Figure 2**). The clinicopathological features of the 2 types of BTT patients are shown in **Table 3**.

**Table 3:** The clinicopathological features of the 2 types of biliary tumor thrombus patients

Variable	Type I (*n* = 69)	Type II (*n* = 133)	*P*
Age	51.3 ± 11.0	52.9 ± 10.2	0.512
Gender (*N*), male:female	53/16	117/16	0.039
AFP (μg/L), < 400: ≥ 400	33/36	76/57	0.208
ALT (U/L), < 40: ≥ 40	30/39	23/110	0.001
Total bilirubin (μmol/L)	25.97 ± 44.1	135.3 ± 123.9	0.001
ALP (U/L)	147.9 ± 73.0	262.5 ± 153.1	0.001
PT (S)	12.1 ± 1.0	12.5 ± 1.3	0.079
Hbs Ag (*N*), positive: negative	62/7	111/22	0.219
HBV-DNA (copies/mL)			0.479
< 1,000	52	106	
≥ 1,000	17	27	
Tumor number, single: multiple	45/24	93/40	0.495
Tumor diameter (cm)	7.34 ± 5.08	5.52 ± 3.35	0.052
Coexistence of PVTT (*n*)	18/69	23/133	0.148
Child-Pugh A:B	65/4	52/81	0.001
Anatomic/local resection	41/28	63/70	0.104

**Figure 3:**
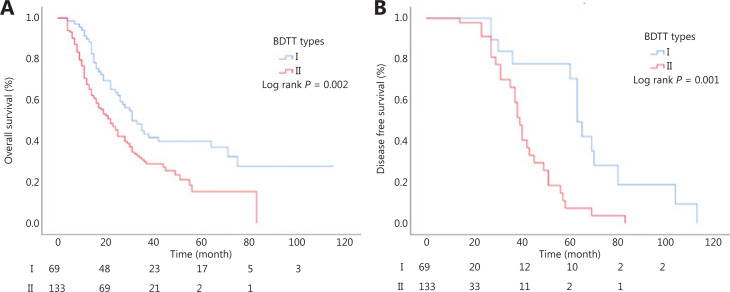
Overall survival according to different biliary tumor thrombus types. (A) The median OS of type I and type II. (B) The median DFS of type I and type II.

The median OS of type I patients was significantly longer than that of type II patients (37.5 months *vs*. 23.2 months; *P* = 0.002) (**Figure 3A**). The median DFS of type I patients was significantly longer than that of type II patients (21.2 months *vs*. 14.0 months, *P* = 0.001) (**Figure 3B**).

### Subgroup analysis

**Figure 4:**
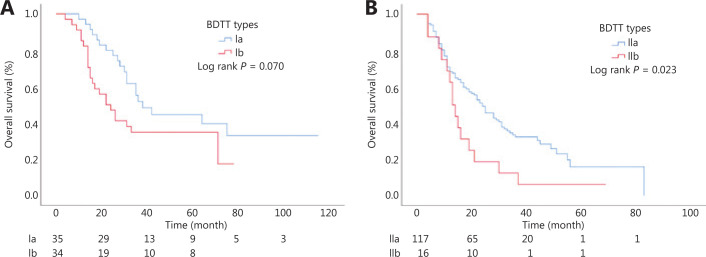
Kaplan-Meier curves of the subtypes of the new biliary tumor thrombus types. (A) The median OS of type Ia and type Ib. (B) The median OS of type IIa and type IIb.

The median OS of the type IIb subgroup was significantly shorter than that of the type IIa patients (17.4 months *vs.* 23.9 months; *P* = 0.023) (**Figure 4A**). Furthermore, if type IIb patients underwent preoperative biliary drainage, the median OS was prolonged from 11.7 months to 22.9 months. There was no significant difference in OS between the type Ia and type Ib BTT groups (44.4 months *vs.* 30.4 months; *P* = 0.070) (**Figure 4B**). The recurrence and survival outcomes of the patients with different subtypes of BTT are shown in **Table 4**.

**Table 4:** Survival outcomes of the 2 biliary tumor thrombus subtypes

Types	Type I: intrahepatic BTT		*P*	Type II: extrahepatic BTT		*P*
Subtypes	Type Ia: tumor thrombosis involving the second-order intrahepatic duct or above	Type Ib: tumor thrombosis involving the first-order intrahepatic duct		Type IIa: TB < 300 μmol/L	Type IIb: TB > 300 μmol/L	
OS (%)			0.070			0.023
1-year	97.1%	82.4%		68.4%	62.5%	
3-year	42.9%	32.4%		24.8%	12.5%	
OS (month)	44.4	30.4		23.9	17.4	

## Discussion

To the best of our knowledge, our study is the first to classify BTT in HCC patients based on both anatomy and TB levels. Multivariate analysis confirmed the new BTT classification is a significant risk factor for OS. This new classification divided BTT into 2 types, which were easy to differentiate by radiological imaging. The new BTT classification is practical and can easily be used in clinical practice. Using this new classification, *en bloc* resection is recommended for HCC patients with type Ia and type Ib BTT. However, for type IIa patients, liver resection combined with thrombectomy should be considered. For type IIb patients, biliary drainage is recommended before surgery.

The incidence of hyperbilirubinemia has been reported to be 19%–40% in HCC patients at the time of diagnosis^13^. Cirrhosis and/or extensive hepatic parenchymal destruction by tumors explains jaundice in the majority of patients, who have a very poor prognosis. With improvements in medical imaging methods^14^, the diagnosis of HCC with BTT can now be made easily. In our data, 92 HCC patients with BTT (45.5%) presented with obstructive jaundice. Identification of this group of patients is clinically important because surgical treatment is usually beneficial^15^. Many studies have confirmed^2,16,17^ that hepatic resection with removal of the BTT is the treatment of choice in selected BTT patients with resectable hepatic tumors, even for those patients presenting with obstructive jaundice.

The low incidence of BTT has led to an insufficient understanding of this complication, and the commonly used international HCC staging systems rarely consider BTT to be an important prognostic factor. The currently available typing methods for BTT are therefore inadequate for this complex problem. The TNM, JIS, and CLIP staging systems all showed overlapping survival curves using statistical analysis, and they are not refined enough to guide treatment or predict prognosis when HCC patients with BTT are still at a resectable stage. The Japanese B-classification and Ueda typing guidelines are the most widely used typing methods for BTT, but they consider only the extent of BTT, without any consideration of the degree of hyperbilirubinemia, which is a very common presentation of BTT, as well as an independent prognostic factor for OS. Thus, our staging system is clinically better for HCC patients with BTT.

Type II BTT patients accounted for approximately 2/3 of all patients in our series, and the treatment of these patients was more complex than that of type I patients. Many clinical problems have not been solved, including when preoperative biliary drainage should be used and how low the TB level should be before liver resection is conducted. Our data showed that TB < 300 μmol/L was an independent prognostic factor of OS, and for patients with TB levels higher than 300 μmol/L, the median OS was significantly prolonged with preoperative biliary drainage. Our study suggested that HCC patients with type IIb BTT should undergo preoperative biliary drainage.

This study had limitations. First, this was a retrospective study with inherent bias. Second, the sample size was small. Third, this study was performed in China with a high percentage of HBV-related HCC patients. Whether the results of this study can be extrapolated to other causes of HCC is unknown. Fourth, all patients in the study underwent liver resection, so, this typing method cannot be used in patients treated without surgery.

In conclusion, a new BTT classification was established. It can be used to predict the prognoses of HCC patients with BTT who underwent partial hepatectomy. This BTT classification is better for predicting prognosis and guiding treatment than other commonly used staging systems. Furthermore, this new classification can also be used to supplement HCC classification or scoring systems.
